# Application of Indole-Alkaloid Harmaline Induces Physical Damage to Photosystem II Antenna Complexes in Adult Plants of *Arabidopsis thaliana* (L.) Heynh

**DOI:** 10.1021/acs.jafc.3c00531

**Published:** 2023-04-07

**Authors:** Sara Álvarez-Rodríguez, Carla M. Alvite, Manuel J. Reigosa, Adela M. Sánchez-Moreiras, Fabrizio Araniti

**Affiliations:** † Departamento de Bioloxía Vexetal e Ciencias do Solo, Facultade de Bioloxía, Universidade de Vigo, Campus Lagoas-Marcosende s/n, 36310, Vigo, Spain; ‡ Dipartimento di Scienze Agrarie e Ambientali - Produzione, Territorio, Agroenergia, Università Statale di Milano, Via Celoria n° 2, 20133 Milano, Italy

**Keywords:** metabolomics, photosynthesis, senescence process, phytotoxicity, herbicide, osmoprotectants

## Abstract

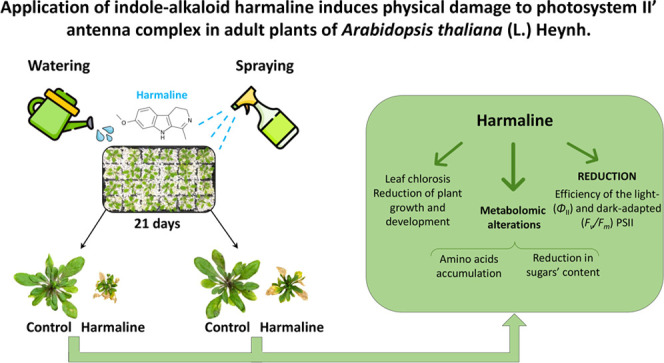

Finding herbicides with new and multiple modes of action is a solution to stop the increase in resistant weed species. Harmaline, a natural alkaloid with proven phytotoxic potential, was tested on *Arabidopsis* adult plants by watering and spraying; watering resulted as the more effective treatment. Harmaline altered several photosynthetic parameters, reducing the efficiency of the light- (Φ_II_) and dark-adapted (*F_v_*/*F_m_*) PSII, suggesting physical damages in photosystem II, although dissipation of the energy in excess under the form of heat was not compromised as demonstrated by the significant increase in Φ_NPQ_. Metabolomic alterations, such as osmoprotectant accumulation and reduction in sugars’ content, also indicate a reduction of photosynthetic efficiency and suggest early senescence and water status alteration induced by harmaline. Data suggest that harmaline might be considered a new phytotoxic molecule interesting for further studies.

## 1 Introduction

The widespread resistance of weeds to commercial synthetic herbicides has increased the demand for alternative molecules (natural or synthetic) with new modes of action (MOAs). Since the 80s, most of the herbicides introduced in the market show old MOAs already determined,^1^ and only one herbicide with a new MOA, cyclopyrimorate, has been commercialized in the last 40 years.^2^ The absence of new herbicides with new MOAs ready for market incorporation is partly due to three economic factors.^3^ The first one is the appearance of glyphosate-resistant crops, which increased farmers’ confidence in this herbicide, adopting glyphosate as the main herbicide for weed management. This led to a devaluation of the other herbicides, which was even stronger when the price of glyphosate went down after patent expiration.^4^ The second factor is the decrease in the number of companies and scientific societies working in herbicide discovery over the past years.^5^ Finally, the third factor is related to the costs of discovering and developing a new herbicide, which is relatively higher than the costs 20 years ago, mainly due to the mandatory evaluation of its environmental impact, which increases the costs.^3^

This situation has favored the massive use of synthetic herbicides and the concomitant increase in resistant weed species, sometimes showing multiresistance to more than one herbicide.^6^ For this reason, new biological agents with phytotoxic potential against weeds as an alternative are being studied to find eco-friendly and botanical-based herbicides with possible new modes of action.

Botanical-based herbicides are molecules that can be derived from plant extracts, sometimes based on the specialized metabolites that plants use to compete with other species for edaphic resources, or to defend themselves from biotic or abiotic stressors.^7^ The group of alkaloids, particularly indole alkaloids, has attracted attention over the past years due to their reported biological activities, such as antiproliferative and antibacterial,^8^ antiviral,^9^ insecticidal,^10^ or antidepressant^11^ activities, among others. In addition, their roles in plants have been widely discussed, and recent studies reported the phytotoxic effects and inhibitory activity of seven alkaloids produced by *Sophora alopecuroides* against weeds.^12^ Nebo et al.^13^ also isolated three alkaloids (evolitrine, kokusagine, and graveoline) from 11 species belonging to Rutaceae and Melicaeae families and found that one of these alkaloids, graveoline, showed a similar phytotoxic potential to the commercial herbicide Logran. Harmaline is also an alkaloid belonging to the group of natural β-carboline alkaloids. This bioactive molecule is mainly found in the plant species *Peganum harmala*,^14^*Banisteriopsis caapi*,^15^ or *Passiflora incarnata* L.^16^ Its phytotoxicity has already been described *in vitro* on *Arabidopsis thaliana* seedlings, pointing out strong aberrations on the anatomy and ultrastructure of the root meristem, mediated by an alteration of the root hormonal balance.^17^ However, only few information is available about the effects of harmaline on adult plants. Although Shao et al.^18^ evaluated harmine and harmaline effects on root and shoot length through inhibitory assays with different crops, such as wheat or lettuce, nothing related to internal changes caused in adult plant metabolism has been studied up to now.

The mode of action of several specialized metabolites with herbicidal activities has already been studied on *A. thaliana* adult plants over the past few years. Stress effects on adult plants have been evaluated through chlorophyll *a* fluorescence analyses, considered a non-invasive and cheap technique that allows fast detection of physiological changes.^19^ For example, *trans-*chalcone,^20^ citral,^21^ coumarin,^22^ or norharmane^23^ showed phytotoxic activity against *A. thaliana* plants, causing different alterations in photosynthetic parameters during treatments when plants were watered or sprayed with the compounds. In addition, metabolomic studies have begun to be widely used to study the mode of action of many natural compounds with phytotoxic potential because they can offer interesting information about plant metabolism alterations.^22,24,25^ This is important since plants modify the levels of many metabolites, such as amino acids, sugars, or other osmolytes, in response to stress conditions.^26^ Abiotic stressors such as salinity, nutrient deficiency/excess, or water deficiency, among others, stimulate the accumulation or dysregulation of metabolic content by increasing osmoprotectant production and accumulation.^27^ For this reason, GC–MS-driven untargeted metabolomic analysis could help to evaluate plant metabolic adjustments and strategies in response to stress.

In this work, morphological, physiological, and metabolomic analyses were done to study the physiological changes induced by harmaline on the adult plant metabolism of the model species *A. thaliana.* This study will help to understand the potential mechanism/s of action of this indole alkaloid on adult plants, forming the basis for the potential development of a new botanical herbicide or for the use of its backbone for the development of new classes of natural-based herbicides.

## 2 Materials and Methods

### 2.1 Plant Growth Conditions

Seeds of *A. thaliana* (L.) Heynh. ecotype Columbia (Col-0) were sterilized in 50% EtOH and 0.5% NaOCl for 3 min each, washed with ultrapure water three times, and preserved in 0.1% agar solution at 4 °C for 72 h. Seeds were then sown in square Petri dishes (120 × 120 mm) using plant agar medium (Duchefa, Holland) with 0.44% of macro- and micronutrients mix (Murashige-Skoog, Sigma-Aldrich, USA) and 1% sucrose. After 15 days in a growth chamber at 22 ± 2 °C, 16 h/8 h day/night, 140 μmol m^–2^ s^–1^ light intensity, and 55% relative humidity, 24 plants with similar size and shape were selected for each treatment and transferred to individual pots containing inert perlite as a substrate. Plants were watered twice a week with 50% Hoagland solution and acclimated for 7 days in a growth chamber with the previously mentioned conditions.

Harmaline (Sigma-Aldrich) was diluted in heated ultrapure water (121 °C) to reach the following concentrations: 14, 28, 56, and 112 μM. All the treatments were applied by watering (sub-irrigation) or spraying. The concentrations were selected based on the IC_50_ value obtained in the *in vitro* harmaline bioassays published by Álvarez-Rodríguez et al.^17^

Trays for watering experiments were irrigated every other day with 350 mL of 50% Hoagland solution, enriched with different harmaline solutions, for 21 days. Trays for spraying experiments were also watered with 350 mL of 50% Hoagland solution every other day for 21 days, but the leaves were daily sprayed with 10 mL of ultrapure water in the case of the control, and 10 mL of each harmaline solution in the case of treatments until the leaves were homogeneously covered with tiny droplets (both the control and treatments were supplemented with 0.001% of Tween-20).

### 2.2 Chlorophyll *a* Fluorescence Measurements

After the first treatment with harmaline, and every two days during 21 days, different chlorophyll *a* fluorescence parameters were recorded using a handheld fluorometer (MultispeQ v2.0, PhotosynQ Inc., East Lansing, MI, USA), according to the Rapid Information Dense Experimental Sequence (RIDES) protocol (“Photosynthesis RIDES 2.0”) available on the PhotosynQ platform [https://photosynq.org/].^28^ First, plants were kept in darkness for 20 min to open all the reaction centers, and maximum quantum efficiency of dark-adapted photosystem II (*F*_v_/*F*_m_) was monitored. Then, the following parameters were also monitored: (i) the effective quantum yield of the photosystem II photochemical reactions (Φ_II_), (ii) the regulated energy dissipation in the form of heat (Φ_NPQ_), and (iii) the nonregulated energy dissipation (Φ_NO_, fluorescence emission).^28^ All measurements were performed *in situ* without damaging the plants, and five plants per treatment were randomly selected per day to obtain five measurements for each parameter at each time.

### 2.3 Post-harvest Measurements

Plants were harvested after 21 days of treatment, and the following parameters were immediately recorded: fresh weight (FW), dry weight (DW), DW/FW ratio, relative water content (RWC), number of leaves, and total leaf area.

#### 2.3.1 Number of Leaves

The number of leaves of five plants per treatment was counted every two days during the 21 days of harmaline treatment for watering and spraying experiments.

#### 2.3.2 Total Leaf Area

All leaves of the three plants were photographed, and the total leaf area was measured for each plant and treatment using the open-source software ImageJ. The results (cm^2^) were expressed as percentage of the control.

#### 2.3.3 Dry/Fresh Weight

Aerial parts of the three plants per treatment were selected, weighed (FW), and over-dried at 70 °C for 72 h. After this time, samples were weighed again (DW), and the data were used to calculate the DW/FW ratio. Data were then expressed as a percentage of the control.

#### 2.3.4 Relative Water Content (RWC)

Three plants per treatment were weighed (fresh weight) and embedded in ultrapure water for 24 h. After this time, samples were weighed again (turgid weight) and over-dried at 70 °C for 72 h to obtain dry weight. RWC was calculated using the following equation: (fresh weight – dry weight)/(turgid weight – dry weight) × 100*.*^29^ Data were then expressed as a percentage of the control.

### 2.4 Extraction, Identification, and Quantification of Primary Metabolites by Metabolomics

#### 2.4.1 Untargeted Metabolomic Analysis

To evaluate harmaline effects on *A. thaliana* adult plants’ metabolism, 24 plants per treatment were treated with 0, 14, 28, 56, and 112 μM harmaline concentrations for 21 days, as previously described. The extraction, derivatization process, and GC–MS analyses were carried out using four replications, as described by Lisec et al.^30^

After harvest, 100 mg of plant material per treatment and replicate was powdered in liquid nitrogen and stored in 2 mL vials.

For the extraction, 1400 μL of cold methanol (−20 °C) was added and vortexed for 10 s. A total of 60 μL of ribitol (0.2 mg mL^–1^ stock in ultrapure H_2_O) was used on each replicate as an internal quantitative standard for the polar phase. Then, samples were shaken for 10 min (950 rpm) in a thermomixer at 70 °C and centrifuged for 10 min at 11000*g*. After that, 1200 μL of supernatant was transferred into glass vials, and 750 μL of CHCl_3_ (−20 °C) and 1500 μL of ultrapure water (4 °C) were sequentially added to each replicate. Vials were carefully vortexed for 10 s and centrifuged at 2200*g* for 15 min to separate the phases. Then, 150 μL of the upper polar phase was transferred into 1.5 mL vials and dried for 2 h and 30 min in a vacuum concentrator without adding heat. After drying, 40 μL of methoxyamine hydrochloride (20 mg mL^–1^ in pyridine) was added for the derivatization of the samples, and samples were incubated in a thermomixer (950 rpm) at 30 °C for 2 h. Then, 70 μL of MSTFA was added to the aliquots, and after shaking at 30 °C for 30 min, 110 μL of each replicate was transferred into the glass vials for GC/MS analysis.

#### 2.4.2 GC-Quadrupole/MS Analysis

A gas chromatograph apparatus (Agilent 789A GC) equipped with a single quadrupole mass spectrometer (Agilent 5975C) was used to inject the extracts already derivatized into a MEGA-5MS capillary column (30 m × 0.25 mm × 0.25 μm equipped with a 10 m pre-column). Temperatures for the source and injector were fixed at 260 and 250 °C, respectively. One μL of each sample was injected with a helium flow of 1 mL min^–1^ following this programmed temperature: isothermal at 70 °C for 5 min followed by a 5 °C/min ramp to 350 °C and a final 5 min heating at 330 °C. Electronic impact (EI) mode at 70 eV was used to record the mass spectra, scanning at 40–600 *m*/*z* range, and using a 0.2 s scan period. The mass spectrometric solvent delay was settled at 9 min. Quality controls (QCs), *n*-alkane standards, and blank solvents (pyridine, methoxyamine hydrochloride, and MSTFA) were injected at scheduled intervals for instrumental performance, tentative identification, and monitoring of shifts in retention indices (RI).

#### 2.4.3 GC/MS Data Analysis Using MS-DIAL

The MS-DIAL with open-source EI spectra library was employed for raw peak extraction, data baseline filtering and calibration, peak alignment, deconvolution analysis, peak identification, and peak height integration. Peak identification was carried out using an average peak width of 20 scans and a minimum peak height of 1000 amplitudes, while deconvolution was carried out using a sigma window value of 0.5 and an EI spectra cutoff of 5000 amplitudes. Retention time tolerance was set at 0.2 min for peak identification, *m*/*z* tolerance at 0.5 Da, EI similarity cutoff at 60%, and identification score cutoff at 80%. During the alignment parameter setting process, the retention time tolerance and the retention time factor were set to 0.5 min. Publicly available libraries, such as the MSRI spectral libraries from Golm Metabolome Database, available from Max Planck Institute for Plant Physiology, Golm, Germany (http://gmd.mpimp-golm.mpg.de/), MassBank, and MoNA (Mass Bank of North America, http://mona.fiehnlab.ucdavis.edu/), were used for MS-DIAL data annotations and compound identification based on the mass spectral pattern.

For metabolite annotation and assignment of the EI-MS spectra, the standard metabolomic initiative (MSI) guidelines for metabolite identification were followed, and chemicals were annotated at level 2 and level 3.^31^

### 2.5 Statistical Analysis

All the experiments were carried out in a completely randomized design using different replicates depending on the experiments (see below). After checking and excluding the outliers detected through Tukey’s method by SPSS Statistics 25.0, data were tested for normality by the Kolmogorov–Smirnov test and heteroscedasticity by Levene’s test. Statistically significant differences between groups were estimated by ANOVA analysis followed by the Tukey’s test as post-hoc in the case of homoscedastic data and Tamhane’s T2 test as post-hoc in the case of heteroscedastic data (*p* ≤ 0.05). Kruskal–Wallis’ test was used for non-normally distributed data and for experiments with *N* = 3.

Metabolomic experiments were carried out using four replications per treatment. The annotated metabolites were analyzed using the Metaboanalyst 5.0 software. The data were normalized using the Lowess-normalization function available in the MS-DIAL software and the internal standard and QCs. After normalization, the missing values were replaced with half of the minimum value found, and then data were Log_10_ transformed and Pareto-scaled. Data groups were discriminated and classified through the principal component analysis (PCA) and the partial least-squares discriminant analysis (PLS-DA), calculating the corresponding variable importance in the projection (VIP value). The one-way analysis of variance (ANOVA) was done using the Tukey’s test as post-hoc (*p* ≤ 0.05). Finally, an enrichment and pathway analysis was conducted using the Metaboanalyst 5.0 tools and setting *A. thaliana* as the metabolome reference database. All the raw and analyzed metabolomic data are reported in Supporting Information, Table S1.

## 3 Results

### 3.1 Harmaline-Induced Inhibition of Growth and Development on *A. thaliana* Plants

**Figure 1:**
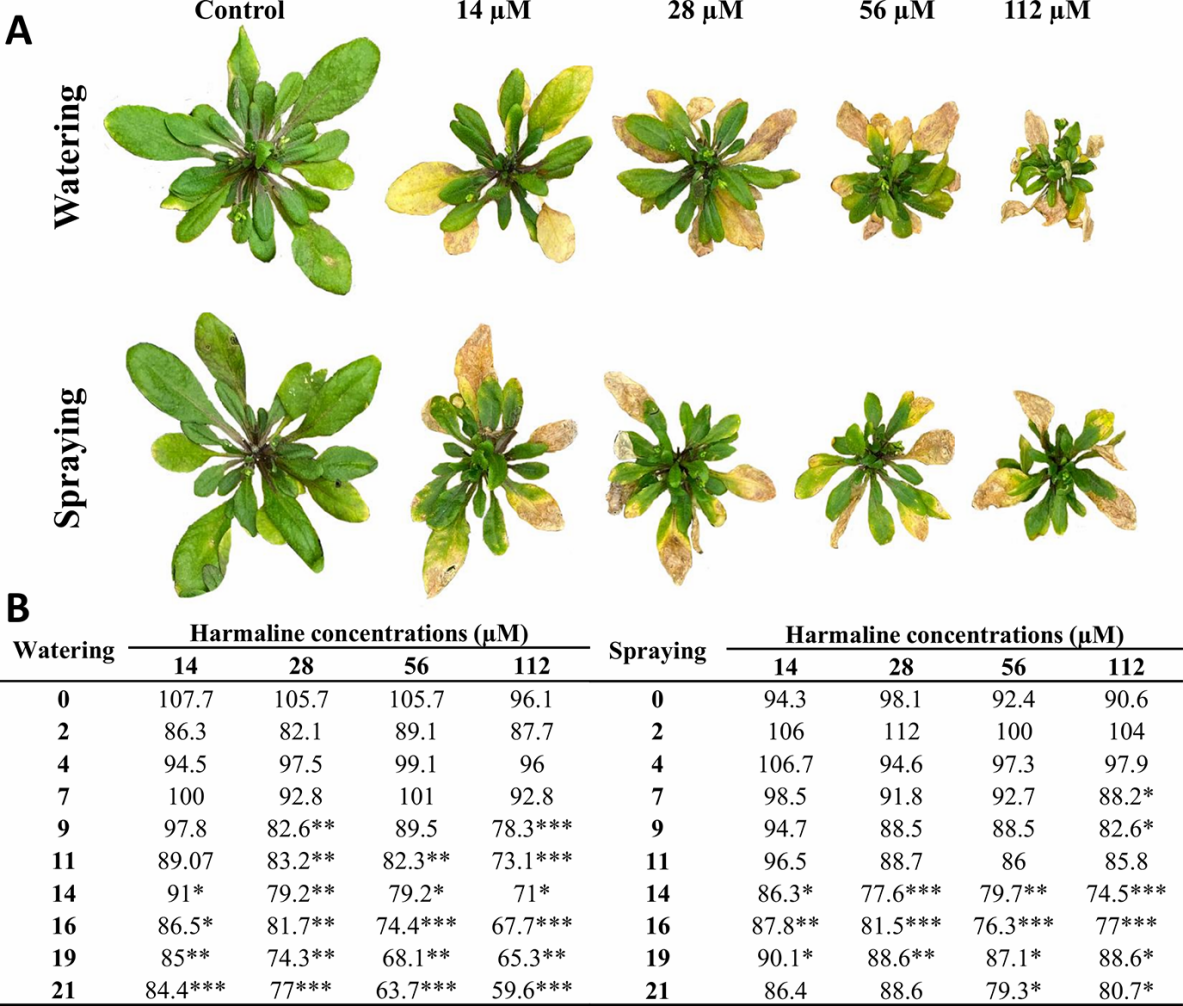
(A) Images of adult plants of *Arabidopsis* watered or sprayed for 21 days with different harmaline concentrations (14, 28, 56, and 112 μM); control plants are shown at the left. (B) Tables with the percentage of number of leaves compared to the control and counted at 0, 2, 4, 7, 9, 11, 14, 16, 19, and 21 days after treatment. Asterisks indicate statistical differences compared to the control. * *p* < 0.05, ** *p* < 0.01, *** *p* < 0.001. *N* = 5.

**Figure 2:**
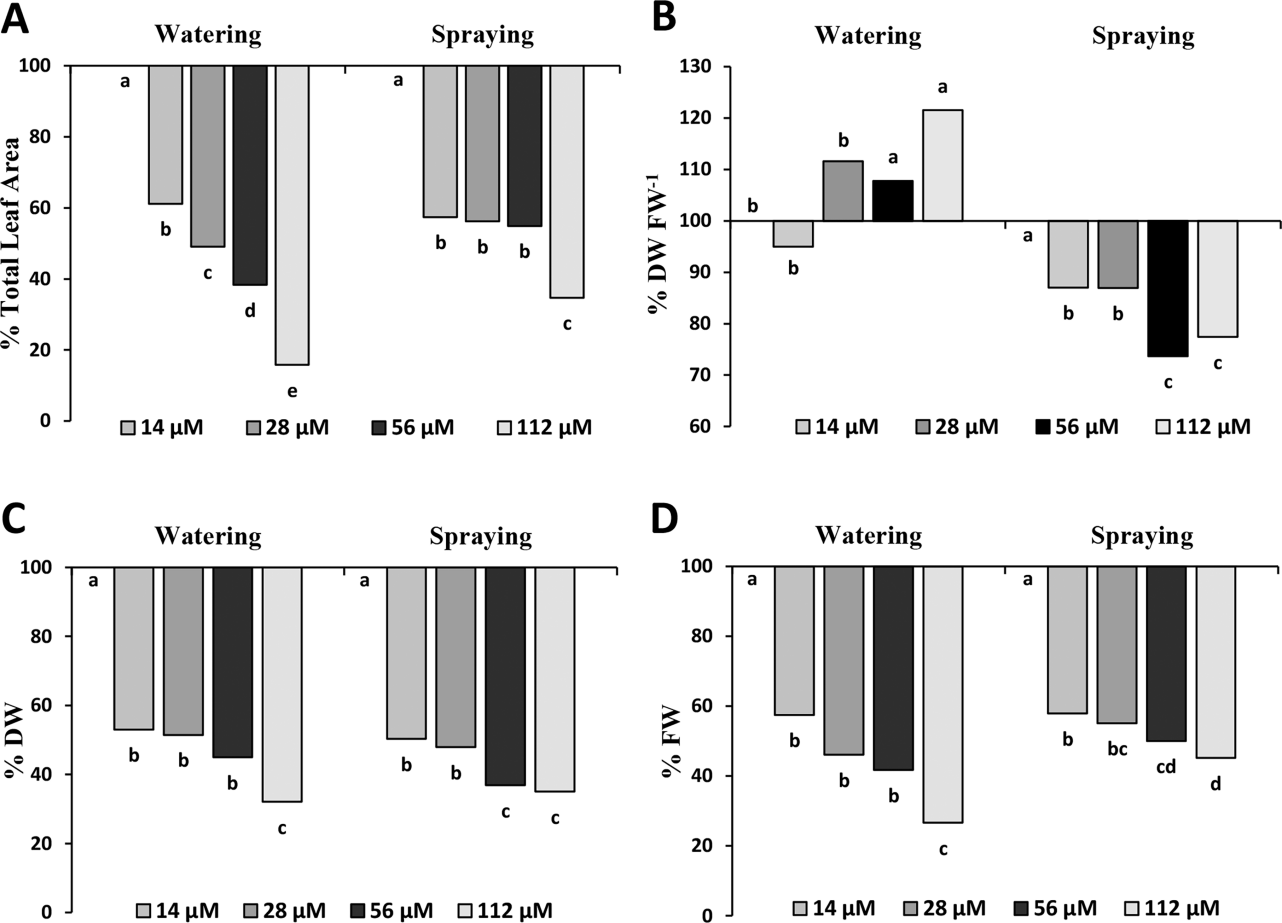
(A) Total leaf area, (B) dry weight/fresh weight ratio (DW/FW), (C) dry weight (DW), and (D) Fresh weight (FW) for harmaline-watered or harmaline-sprayed plants. All data are given in percentage of the control, with the control treatment expressed on the *x*-axis as 100%. Different letters indicate statistical differences between treatments (*p* < 0.05). *N* = 3.

Harmaline-watered *Arabidopsis* plants showed strong inhibition of plant growth due to reduced leaf number and area (Figure 1A,B; Figure 2A). In addition, a significant difference in leaf number was already observable at the highest concentration assayed (112 μM) after 9 days of treatment and at all the concentrations assayed after 14 days of harmaline treatment. Plants watered with the lowest concentration of harmaline (14 μM) showed a 40% reduction of leaf area at the end of the experiment, which was 50, 62, and 85% of the control for the harmaline treatments 28, 56, and 112 μM, respectively (Figure 2A). In addition, harmaline-watered plants were characterized by the presence of chlorotic and necrotic areas, especially in older leaves, after 21 days of treatment (Figure 1A).

Similar to watered-treated plants, harmaline-sprayed plants were also significantly smaller than control plants (Figure 1A). The total leaf area significantly decreased by 50% for 14, 28, and 56 μM harmaline-sprayed plants (Figure 2A) and up to 65% for the highest concentration sprayed (112 μM). Regarding leaf number, significant reductions of 10 to 25% of the control were obtained for all concentrations assayed from day 14 of harmaline spraying. As also observed during watering treatment, harmaline-sprayed leaves showed chlorotic and necrotic areas (Figure 1A).

Fresh (FW) and dry (DW) weight of treated plants confirmed the damage of harmaline treatment. Both FW and DW showed dose-dependent reductions. In particular, FW decreased between 40 and 75% for watering and 40 and 55% for spraying. The strongest watering treatment (112 μM) was the most phytotoxic one, inducing a reduction of 75% in fresh biomass production (Figure 2D). Dry weight values were also similar during watering and spraying applications, reaching a significant biomass reduction of about 55–70% for both application techniques (Figure 2C). On the contrary, the DW/FW ratio was differentially affected by the two application treatments. In particular, the ratio was significantly increased in harmaline-watered plants and significantly decreased in sprayed plants compared to the control (Figure 2B). Finally, the RWC was unaffected in harmaline-watered plants, although a slight but significant increase was observed in 56 μM harmaline-sprayed plants (Figure S1).

### 3.2 Photosynthetic Alteration on *Arabidopsis* Plants after Harmaline Treatment

**Figure 3:**
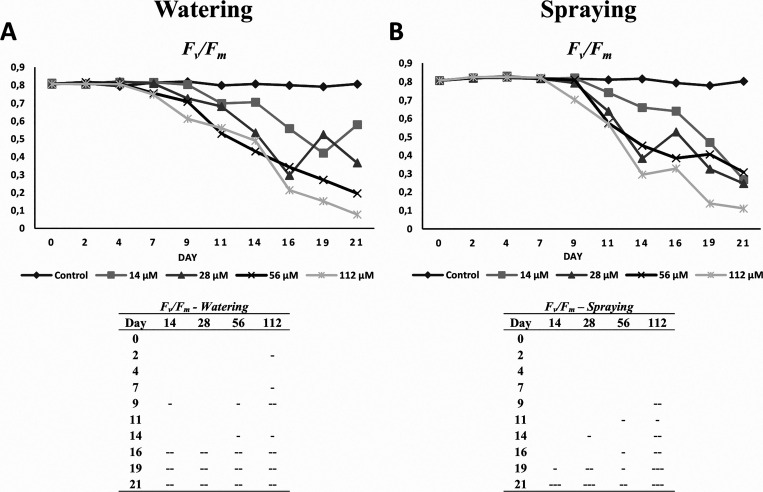
Mean values of *F_v_*/*F_m_* in plants (A) watered or (B) sprayed for 21 days with 14, 28, 56, and 112 μM harmaline. Tables show the statistical significance differences compared to untreated plants (+, positive difference; -, negative difference; + or -, *p* < 0.05, ++ or --, *p* < 0.01, +++ or ---, *p* < 0.001). Mean values of *F_v_*/*F_m_* are expressed in arbitrary units (AU). *N* = 5.

**Figure 4:**
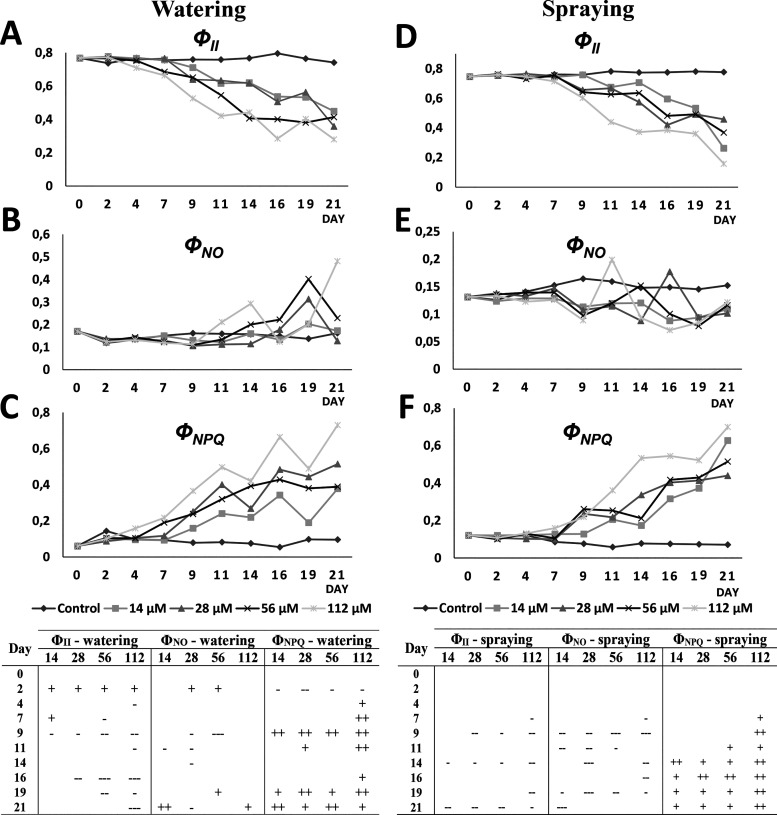
Mean values of Φ_II_ (PSII efficiency), Φ_NO_ (fluorescence emission), and Φ_NPQ_ (non-photochemical quenching) in plants (A–C, respectively) watered or (D–F, respectively) sprayed for 21 days with 14, 28, 56, and 112 μM harmaline. Tables show the statistical significance differences compared to untreated plants (+, positive difference; -, negative difference; + or -, *p* < 0.05, ++ or --, *p* < 0.01, +++ or ---, *p* < 0.001). Mean values of Φ_II_, Φ_NO_, and Φ_NPQ_ are expressed in arbitrary units (AU). *N* = 5.

Regarding the photosynthetic activity of harmaline-treated *Arabidopsis* plants, both photochemical and non-photochemical pathways of harmaline-watered and sprayed plants were significantly altered, especially *F_v_*/*F_m_* (Figure 3), but also Φ_II_, Φ_NPQ_, and Φ_NO_ (Figure 4). Although similar effects (reduction in *F_v_*/*F_m_* and *Φ*_II_ and increase in Φ_NPQ_) were observed for both harmaline-watered and sprayed plants, the damage was earlier detectable in harmaline-watered plants.

The maximum PSII efficiency (*F_v_*/*F_m_* ratio) showed a downward trend for both harmaline application treatments. In general, *F_v_*/*F_m_* was not affected in harmaline-sprayed and watered plants during the first seven days of treatment. However, while in harmaline-watered plants, *F_v_*/*F_m_* values started to decrease from day 9 for the higher concentrations tested (56 and 112 μM; Figure 3A) and from day 16 for all the concentrations, in harmaline-sprayed plants, *F_v_*/*F_m_* values started to decrease from day 9 just for the highest concentration tested (112 μM; Figure 3A) and from day 19 for all the concentrations (Figure 3B).

Regarding the efficiency of PSII (Φ_II_), a significant reduction in plants watered with all concentrations of harmaline from the second day of treatment was observed (Figure 4A). The values fluctuated until day 9 of treatment when Φ_II_ significantly decreased for all harmaline concentrations. Plants watered with 112 μM showed a significant reduction of Φ_II_ from day 16 to the end of the study, while for 28 and 56 μM, Φ_II_ was only significantly reduced at days 16 and 19, respectively. However, Φ_II_ values of plants sprayed with the strongest concentration of harmaline (112 μM) did not start to decrease until day 7, and reductions of Φ_II_ were significant in all concentrations for days 14 and 21 of treatment (Figure 4D). However, there was a constant downward trend in Φ_II_ values from day 9 to the end of the experiment.

In both watering and spraying treatments, reduced Φ_II_ values resulted in increased Φ_NPQ_ values (regulated emission of the energy in excess in the form of heat). Already on the second day of treatment, harmaline-watered plants showed Φ_NPQ_ values significantly higher than the control for all the concentrations tested (Figure 4C). Plants watered with 112 μM harmaline showed a constant and significant increase in heat emission from the beginning to the end of the experiment. However, lower harmaline concentrations reached significantly increased levels of Φ_NPQ_ compared to untreated plants on day 9 and for the last two days of treatment, 19 and 21. Concerning harmaline-sprayed plants, the behavior of the Φ_NPQ_ parameter was similar to that of the watered plants, but the effect was later and more homogeneous among treatments (Figure 4F). Significant increase of heat dissipation were observed with 112 μM harmaline after one week and for 56 μM after 9 days of treatment. From day 14 to the end of the spraying experiment, Φ_NPQ_ was significantly higher than the control for all the concentrations tested.

On the contrary, Φ_NO_ values (fluorescence emission) were not constant along the experiment and fluctuated during watering and spraying treatments, showing more erratic behavior and less significant differences than Φ_II_ and Φ_NPQ_. In general, the emission of chlorophyll *a* fluorescence tended to increase in harmaline-watered plants and decrease in sprayed plants (Figure 4B and Figure 4E, respectively). In 28 and 56 μM harmaline-watered plants, Φ_NO_ significantly increased after 2 days and decreased after 9 days of treatment. However, the trend of Φ_NO_ in harmaline-sprayed plants was characterized by a constant reduction, leading to significantly lower Φ_NO_ values at days 9 and 19 of treatment for all concentrations tested. Despite fluctuations, Φ_NO_ values showed significant reductions for some of the harmaline concentrations during the 21 days of experiment (Figure 4E).

### 3.3 Effects of Harmaline Treatment on Primary Metabolism

The GC–MS-driven untargeted-metabolomic analysis allowed us to putatively annotate and relatively quantify 137 metabolites in watering and 121 in spraying treatment, whereas 482 and 447 unknown metabolites were found in watering and spraying treatments, respectively (Table S1).

System suitability and model robustness were demonstrated by carrying out the Unsupervised Principal Component Analysis (PCA) on QC and sample groups. The PCA Score Plot, built on the first (PC1) and the second component (PC2), showed a clear separation between qualitative controls and sample groups in both watering and spraying treatments (Figure S2A,C). These results suggested that the analytical steps were reliable, reflecting the metabolomic profile changes in *Arabidopsis* adult plants after harmaline treatment. In addition, the PCA analysis on watered and sprayed samples, including known and unknown compounds, confirmed a clear discrimination among the treatments in both experiments (Figure S2B,D).

The univariate one-way analysis of variance (ANOVA) revealed that 119 out of 137 and 108 out of the 121 annotated metabolites were significantly altered in harmaline-watered and sprayed treatments, respectively (the complete list of the significantly altered metabolites and the ANOVA results is available in Table S1). Those 119 and 108 compounds were reported on heatmaps, giving a global view of the trend of each metabolite between harmaline concentrations and treatment techniques (Supporting Information, Figure S4 and Table S1).

**Figure 5:**
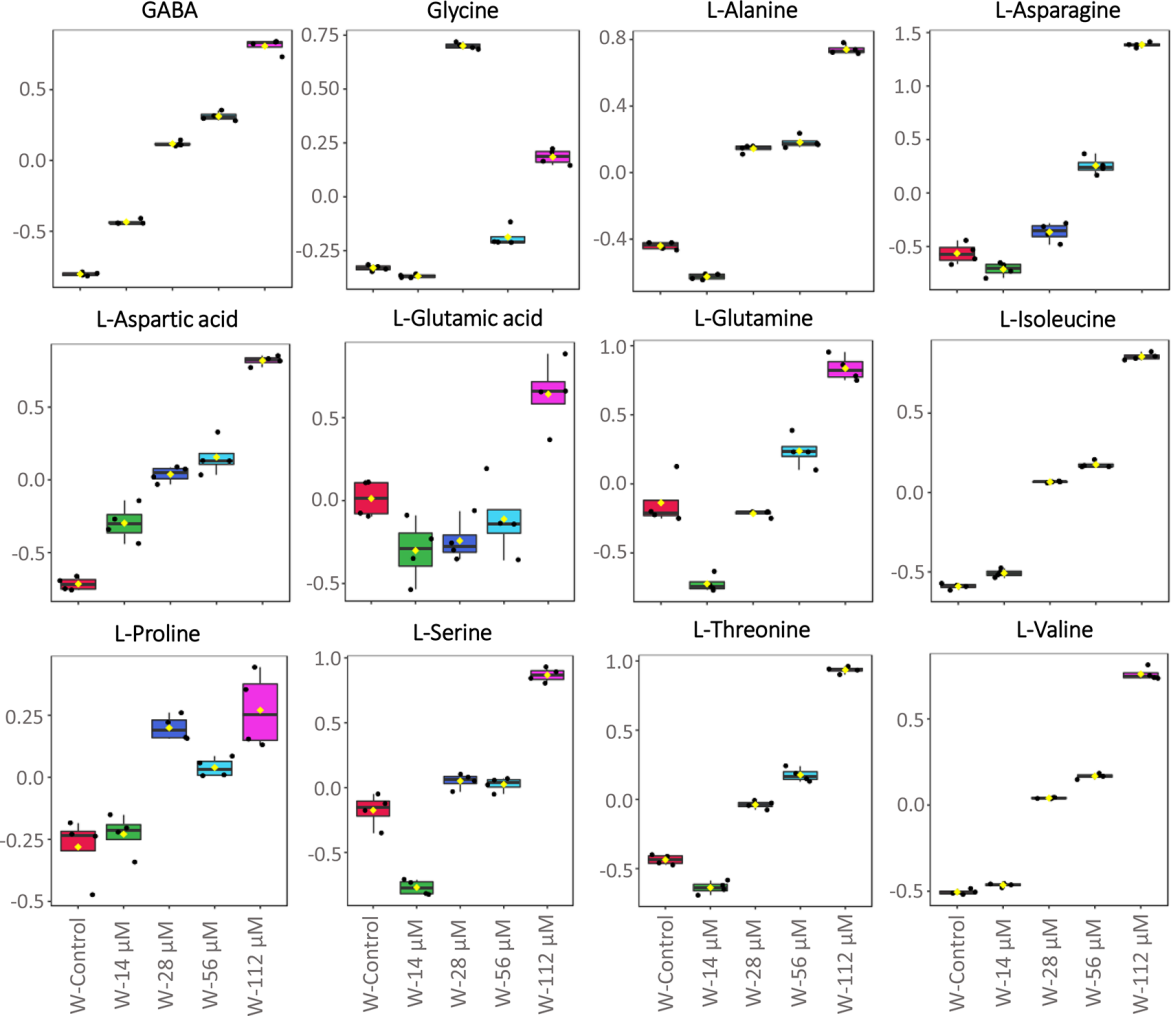
Effects of harmaline watering treatment for 21 days on amino acid contents in *Arabidopsis* adult plants. W-control (watering control; red color), W-14 μM (14 μM harmaline-watered; green color), W-28 μM (28 μM harmaline-watered; dark blue color), W-56 μM (56 μM harmaline-watered; light blue color), W-112 μM (112 μM harmaline-watered; pink color). Normalized metabolomic data were analyzed through ANOVA using the Tukey test as post-hoc (*p* ≤ 0.05). *N* = 4. The full list of the significantly altered metabolites is available in Table S1.

Concerning watering treatment, the most significantly increased organic acids compared to the control were citric, gluconic, glutaric, and uric acids (the last one only at the highest concentration tested). Only succinic acid was lower than that in untreated plants at all the concentrations tested, whereas malic acid was reduced only at 112 μM (Figure S5). Concerning the amino acid content, an upward accumulation trend in treated plants was observed. Some of them (alanine, GABA, l-aspartic acid, l-isoleucine, l-proline, and l-valine) significantly increased in plants treated at all the concentrations assayed, whereas others increased their abundance at concentrations higher than 14 μM (l-alanine, glycine, l-asparagine, l-serine, and l-threonine). Finally, compounds such as l-glutamic acid and l-glutamine significantly increased only at the highest concentrations assayed (Figure 5).

**Figure 6:**
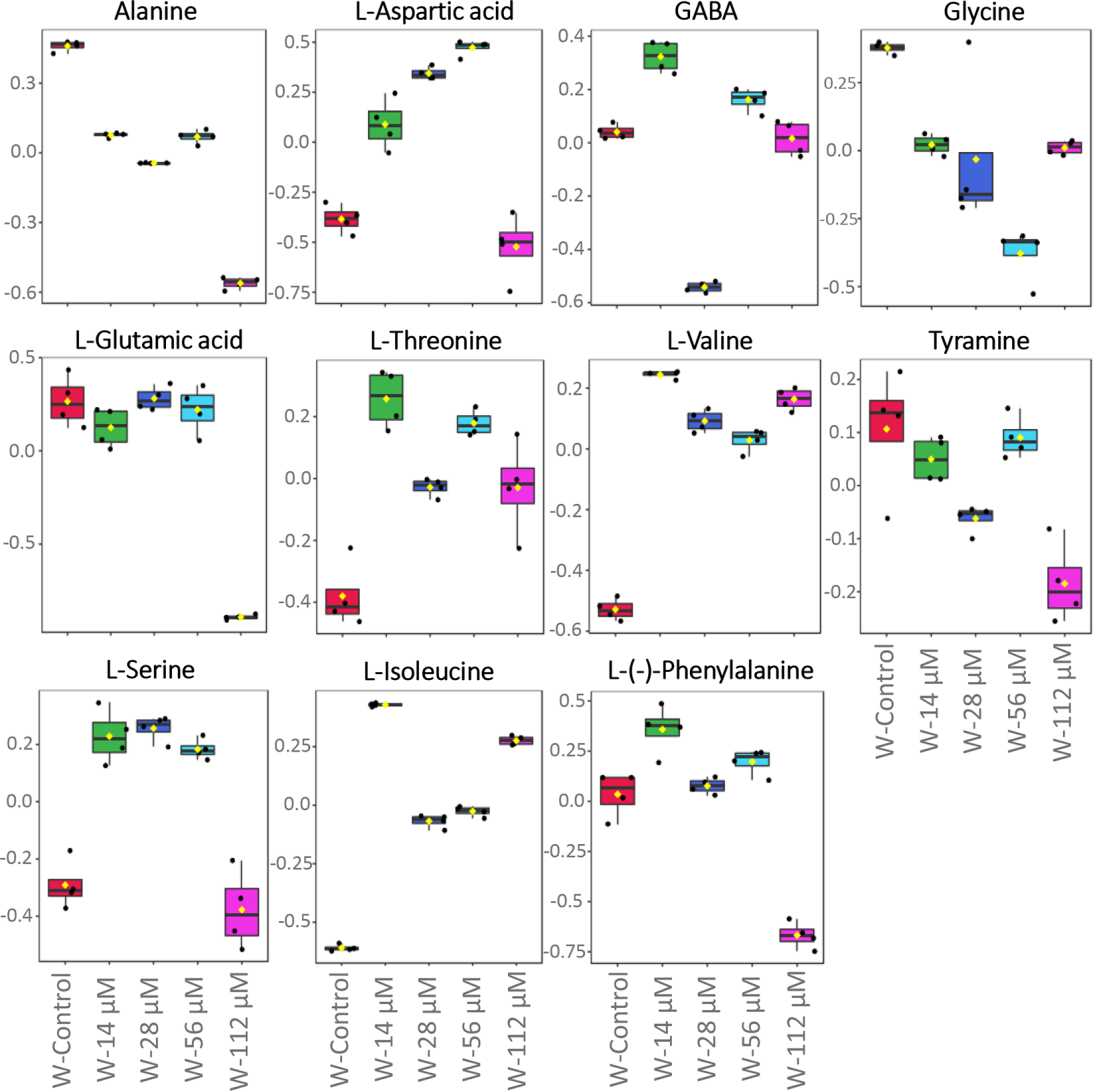
Effects of harmaline spraying treatment for 21 days on amino acid contents in *Arabidopsis* adult plants. S-control (spraying control; red color), S-14 μM (14 μM harmaline-sprayed; green color), S-28 μM (28 μM harmaline-sprayed; dark blue color), S-56 μM (56 μM harmaline-sprayed; light blue color), S-112 μM (112 μM harmaline-sprayed; pink color). Normalized metabolomic data were analyzed through ANOVA using the Tukey test as post-hoc (*p* ≤ 0.05). *N* = 4. The full list of the significantly altered metabolites is available in Table S1.

On the other hand, spraying treatment also increased most of the organic acids’ content, such as aconitic, citric, gluconic, and glutaric acids. However, glyceric or succinic acids were significantly reduced after harmaline spraying treatment (Figure S6). It should be highlighted that the amino acids l-isoleucine, l-threonine, and l-valine increased their levels after harmaline treatment at all the concentrations tested, whereas alanine, glycine, and tyramine were down-accumulated in treated plants. In addition, levels of amino acids like L-glutamic acid or l-phenylalanine significantly dropped at the highest concentration (Figure 6).

**Figure 7:**
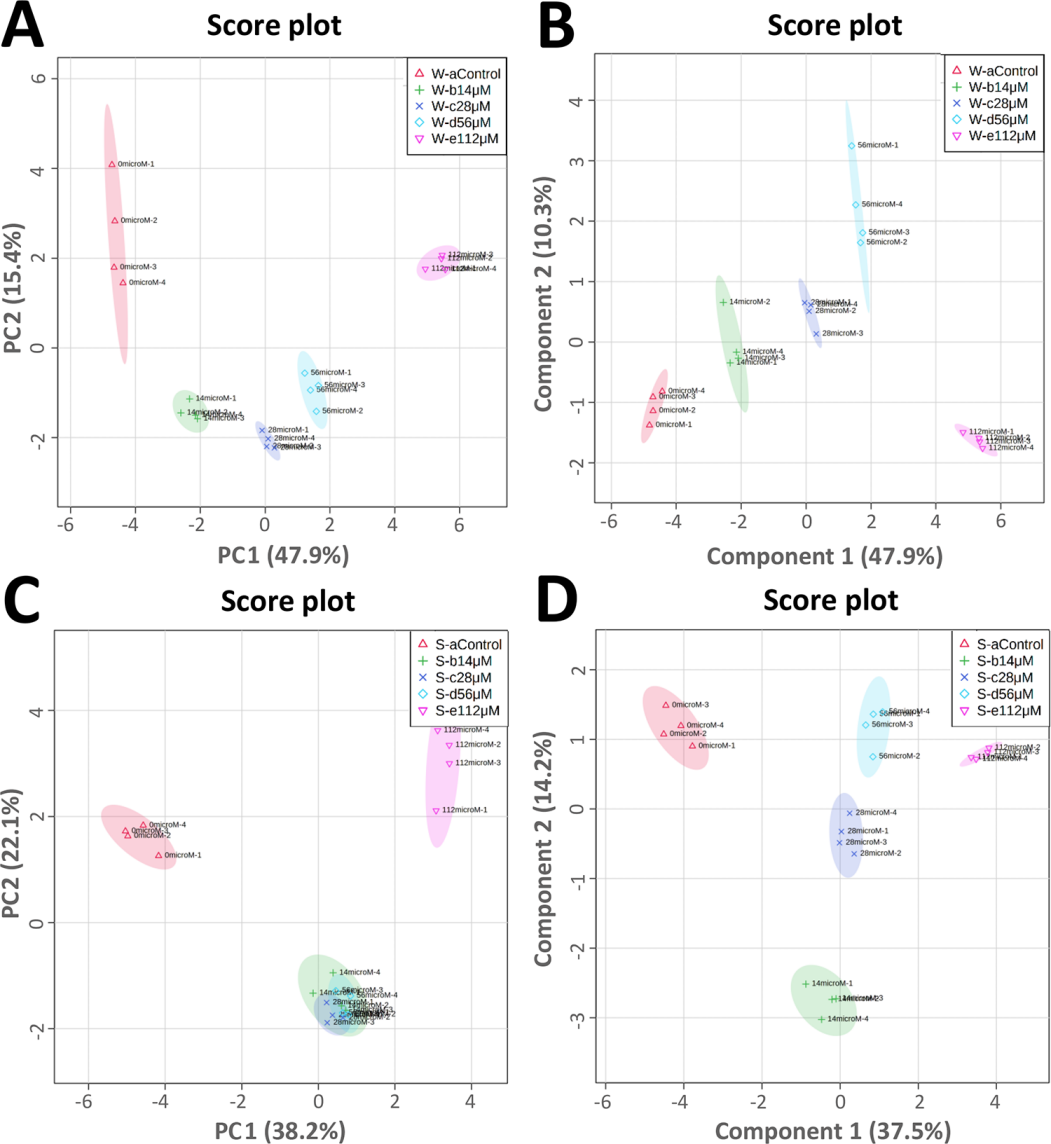
Discrimination through principal component analysis (PCA) and partial least-squares discriminant analysis (PLS-DA) of the metabolites’ patterns in *A. thaliana* adult plants exposed for 21 days to 0, 14, 28, 56, and 112 μM harmaline by watering and spraying. W-acontrol (watering control; red color), S-acontrol (spraying control; red color), W-b14μM (14 μM harmaline-watered; green color), S-b14μM (14 μM harmaline-sprayed; green color), W-c28μM (28 μM harmaline-watered; dark blue color), S-c28μM (28 μM harmaline-sprayed; dark blue color), W-d56μM (56 μM harmaline-watered; light blue color), S-d56μM (56 μM harmaline-sprayed; light blue color), W-e112μM (112 μM harmaline-watered; pink color), and S-e112μM (112 μM harmaline-sprayed; pink color). (A, C) PCA of watering and spraying, respectively, and (B, D) PLS-DA of watering and spraying, respectively, that allowed sample group discrimination by virtue of the first two principal components (PCs). *N* = 4.

The PCA analyses on both watering and spraying treatments were performed by virtue of the first two principal components PCs (PC1 vs PC2). The PC1 represented 47.9 and 38.2% of the total variance in the case of watering and spraying, respectively, whereas PC2 accounted for 15.4 and 22.1%. The analyses highlighted a clear separation between untreated and harmaline-watered plants, whereas in sprayed plants, this separation was observed only at the highest concentration assayed (112 μM) (Figure 7A,C).

The evaluation of the PCA loading plot in harmaline-watered plants highlighted that PC1 was dominated by the metabolites l-asparagine, alanine, pryrrole-2-carboxylic acid, glycerol-alpha-phosphate, and l-threonine, among others, whereas PC2 was mainly dominated by nonadecane, d-(−)-arabinose, lactic acid, octadecanoate, and d-(+)-xylose, among others. On the other hand, PC1 in harmaline-sprayed plants was dominated by *n*-acetyl-d-hexosamine, d-(+)-maltose, 3-aminopropionitrile, 16-methylheptadecanoic acid methyl ester, and citraconic acid, whereas PC2 was dominated by sinigrin, alpha-lactose, l-aspartic acid, 3-ethyl-2,6,10-trimethylundecane, and fructose-6-phosphate (Table S1).

**Figure 8:**
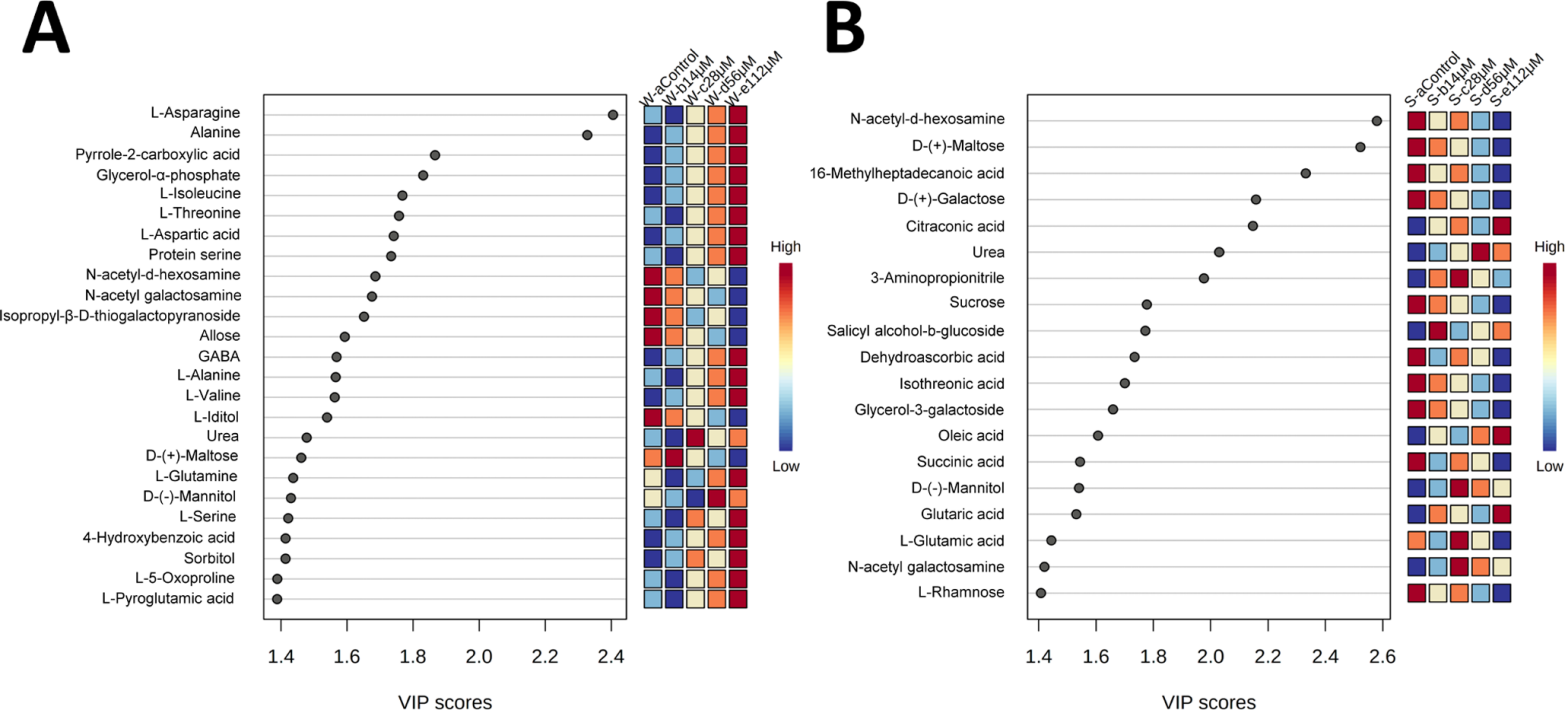
Variable importance of projection (VIP) features with a VIP score higher than 1.4 for the (A) watering and (B) spraying treatment form PLS-DA analysis. W-acontrol (watering control), S-acontrol (spraying control), W-b14μM (14 μM harmaline-watered), S-b14μM (14 μM harmaline-sprayed), W-c28μM (28 μM harmaline-watered), S-c28μM (28 μM harmaline-sprayed), W-d56μM (56 μM harmaline-watered), S-d56μM (56 μM harmaline-sprayed), W-e112μM (112 μM harmaline-watered), S-e112μM (112 μM harmaline-sprayed). *N* = 4.

Also, the PLS-DA analysis carried out on both harmaline treatments further exacerbated the separation among sample groups for both watering and spraying treatments (Figure 7B,D, respectively). The PLS-DA model was built using the first four components explaining a total variance equal to 73.9% in the case of harmaline-watered treatment and 75.1% in the case of harmaline-sprayed plants. The model was further validated through cross-validation and permutation tests, which proved the PLS-DA model’s robustness, reaching high *R*^2^ and *Q*^2^ values for both harmaline applications (*p* ≤ 0.05; Figure S3). The variable importance in projection (VIP) scores (built on the metabolites with a VIP score higher than 1.4) revealed that l-asparagine, alanine, pyrrole-2-carboxylic acid, l-isoleucine, and l-threonine, among others, had the highest VIP scores for harmaline-watered plants (Figure 8A, Table S1). In the case of harmaline-sprayed plants, metabolites with the highest VIP scores in PLS-DA were *n*-acetyl-*d*-hexosamine, d-(+)-maltose, 16-methylheptadecanoic acid methyl ester, d-(+)-galactose, and citraconic acid, among others (Figure 8B, Table S1).

Finally, a KEGG-based pathway analysis was carried out using the metaboanalyst module “MetPa” to evaluate which pathway was affected by harmaline treatments. The pathway analysis revealed an important impact of harmaline on *Arabidopsis* metabolism. In particular, harmaline treatment affected 33 and 34 pathways during watered and sprayed treatments, respectively. The three routes with a pathway impact score higher than 0.5 were alanine, aspartate, and glutamate metabolism, glycine, serine, and threonine metabolism, and C5-branched dibasic acid metabolism, coinciding for both watering and spraying treatments (Table 1 and Table S1). However, alanine, aspartate, and glutamate metabolism, for example, showed a higher impact for harmaline-watered plants than for sprayed plants (0.85 vs 0.65, respectively) (Table 1).

**Table 1:** Results from Ingenuity Pathway Analysis with MetPa Carried out on *Arabidopsis* Adult Plants Watered or Sprayed with 0, 14, 28, 56, and 112 μM Harmaline for 21 Days^a^

Watering							
			14 μM	28 μM	56 μM	112 μM	
pathways	Total Cmpd	hits	raw *p*	raw *p*	raw *p*	raw *p*	impact
alanine, aspartate, and glutamate metabolism	22	8	4.15 × 10^–7^	3.40 × 10^–8^	9.84 × 10^–9^	2.67 × 10^–9^	0.85
glycine, serine, and threonine metabolism	33	4	3.77 × 10^–6^	1.66 × 10^–6^	2.12 × 10^–5^	1.08 × 10^–8^	0.51
C5-branched dibasic acid metabolism	6	1	0.03718	//	//	1.92 × 10^–5^	0.50
glyoxylate and dicarboxylate metabolism	29	10	3.93 × 10^–7^	1.38 × 10^–6^	5.99 × 10^–11^	2.02 × 10^–12^	0.39
beta-alanine metabolism	18	4	0.00040128	1.21 × 10^–6^	3.29 × 10^–6^	7.82 × 10^–9^	0.33
citrate cycle (TCA cycle)	20	5	8.49 × 10^–9^	1.36 × 10^–9^	2.25 × 10^–9^	2.13 × 10^–9^	0.29
starch and sucrose metabolism	22	4	//	//	//	0.019057	0.28
pentose and glucuronate interconversions	16	3	1.33 × 10^–9^	7.08 × 10^–9^	2.31 × 10^–8^	8.16 × 10^–7^	0.22

Spraying							
			14 μM	28 μM	56 μM	112 μM	
pathways	Total Cmpd	hits	raw *p*	raw *p*	raw *p*	raw *p*	impact
alanine, aspartate, and glutamate metabolism	22	6	7.90 × 10^–8^	3.57 × 10^–10^	2.19 × 10^–10^	3.57 × 10^–7^	0.65
glycine, serine, and threonine metabolism	33	5	3.22 × 10^–5^	9.61 × 10^–6^	7.10 × 10^–7^	1.33 × 10^–6^	0.54
C5-branched dibasic acid metabolism	6	1	0.013274	2.42 × 10^–6^	0.016946	3.99 × 10^–7^	0.50
phenylalanine metabolism	11	1	0.0076381	//	0.043982	3.48 × 10^–5^	0.47
glyoxylate and dicarboxylate metabolism	29	9	2.08 × 10^–8^	2.12 × 10^–8^	4.40 × 10^–8^	3.53 × 10^–10^	0.35
beta-alanine metabolism	18	4	2.96 × 10^–5^	5.55 × 10^–9^	1.33 × 10^–8^	8.59 × 10^–6^	0.33
citrate cycle (TCA cycle)	20	5	6.57 × 10^–8^	1.15 × 10^–8^	1.44 × 10^–8^	6.61 × 10^–8^	0.30
starch and sucrose metabolism	22	5	0.041569	0.0026516	0.00054962	0.00026171	0.29
pentose and glucuronate interconversions	16	3	9.52 × 10^–8^	4.07 × 10^–8^	7.03 × 10^–6^	5.20 × 10^–6^	0.22

a Total Cmpd: the total number of compounds in the pathway; hits: the matched number from the uploaded data; raw *p*: the original *p* value calculated from the enrichment analysis; FDR: false discovery rate. Only the pathways with an impact score higher than 0.2 were reported. The “//” indicates not significant differences because *p* ≥ 0.05. The full list of the significantly altered pathways is available in Table S1.

## 4 Discussion

The results of this study demonstrated the strong phytotoxic potential of the indole-alkaloid harmaline on adult plants of *A. thaliana* both for watering and spraying treatments. The results highlighted and confirmed *in situ* the strong *in vitro* phytotoxic potential of this natural molecule on *A. thaliana* metabolism.^17^ In particular, both watering and spraying treatments caused evident phytotoxic effects already at very low concentrations if compared to other phytotoxic molecules reported in the literature.^20−23^ Moreover, the results suggest that harmaline could act by inducing an alteration of the plant-water status followed by the altered redox status with the consequent physical damage to the photosynthetic machinery, affecting plant growth and development.

Clear morphological differences were observed after both harmaline treatments, although the effects of watering on the post-harvest parameters were stronger. In fact, harmaline-watered plants experienced a further reduction in the number of leaves and the size of rosettes than sprayed plants. On the other hand, a strong decrease in FW and DW values and foliar damage such as chlorosis, considered a senescence symptom,^32^ were found in both harmaline-watered and sprayed plants. Similar effects have been reported in other studies with *A. thaliana* adult plants treated with natural compounds such as citral or *trans*-caryophyllene.^21,33^

Contrarily, while the DW/FW ratio increased in harmaline-watered plants, decreased values were obtained for sprayed plants. Water status alteration was also suggested to cause relevant increases in the DW/FW ratio after *Origanum vulgare* essential oil,^34^*trans*-chalcone,^20^*trans*-caryophyllene,^33^ or norharmane^23^ treatments on *A. thaliana* plants. Conversely, the DW/FW ratio was reduced after coumarin treatment when compared to *Arabidopsis* control plants.^22^

The fluorometer was used to monitor possible alterations in the photosynthetic system, and both watered and sprayed plants experienced global and similar changes: reduction in the photochemical quenching (Φ_II_), a constant increase in regulated energy dissipation (Φ_NPQ_), and a significant decrease in maximum PSII efficiency in darkness (*F_v_*/*F_m_*).

Two days after starting the experiment, Φ_II_ and Φ_NO_ significantly increased, while Φ_NPQ_ was significantly lower than control plants in watering treatment. This situation, commonly related to compensation photosynthesis, suggests that the plant was under stress by harmaline. The photosynthetic activity would increase to respond to the energy demand required to face the stress by synthesizing stress-related metabolites and solve the imminent damage due to the inefficiency of treated plants to promptly activate the protective regulatory strategies. Consequently, the first response to the toxicity was an increase in the non-regulated emission of fluorescence (increase in Φ_NO_). However, once the plants activate the regulatory mechanisms of energy dissipation, these mechanisms reduce the fluorescence-related damage and protect the plants, as observed during the rest of the experiment. Lopes et al.^35^ have already observed this situation, suggesting that increases in fluorescence emission can occur without reducing the PSII quantum yield in some types of stresses.

The strong downward *F_v_*/*F_m_* trend, observed in both watering and spraying treatments from day 9 of the experiment, suggests physical damage at the protein-pigment complexes of the light-harvesting antennae of PSII.^19^ Similar results were also observed in plants treated with other natural compounds such as citral, *trans*-chalcone, or *trans*-caryophyllene.^20,21,33^ This effect was associated with a reduction in the photosynthetic efficiency (Φ_II_) and an increase in Φ_NPQ_ values compared to the control. Regulation of Φ_NPQ_ is mediated through three main components: the proton gradient across the thylakoid membrane,^36^ the activity of the xanthophylls cycle,^37^ and the protein PsbS homologous to the antenna components.^38^ This coefficient increase could suggest that harmaline physically damages the PSII and that plants are trying to face this stressful condition through regulated strategies (increase in Φ_NPQ_).

In harmaline-sprayed plants, this protective response was enough to compensate for harmaline-induced damages since the energy emitted in the form of fluorescence (Φ_NO_) was lower than the energy dissipated in the form of heat (Φ_NPQ_) from day 9 till the end of the experiment. However, in harmaline-watered plants, this strategy was not enough to regulate the excess of energy, and harmful emission of fluorescence increased (Φ_NO_) at the end of the experiment. Since all these significant alterations caused by harmaline began to change steadily for both parameters from day 9, these changes could be considered among the primary effects of the compound on plant metabolism. The increase in Φ_NO_ values in harmaline-watered plants could be related to an altered water status of these plants. This hypothesis is supported by the increase in the DW/FW ratio previously observed with other natural compounds such as *trans*-chalcone^20^ or *trans*-caryophyllene,^33^ which induced water status alteration and alterations to the photosynthetic machinery.

Harmaline-treated plants experienced early senescence mainly in the older leaves. In particular, leaf depigmentation and death were already detected after 7 days for the lowest concentration of watering and after 9 days for the lowest concentration of spraying. Mobilizing nutrients from senescent to young leaves in stressed plants is systematically done through metabolic, spatial, and temporal adjustments to protect the young leaves from induced senescence.^39^ Therefore, the measurement of chlorophyll *a* fluorescence, which was done in undamaged leaves (i.e., green young leaves) could be influenced by nutrient mobilization, showing a higher capacity to dissipate the excess of energy in the form of heat instead of fluorescence owing to this protection.

Concerning metabolomic analysis, significant alterations of several pathways belonging to the primary metabolism highlighted an upward trend in organic and amino acid concentrations and a decrease in sugar content.

In harmaline-watered and sprayed plants, a significant increase in isoleucine and valine content was observed. The accumulation of these branched-chain amino acids is generally induced during osmotic stress, and the biosynthesis of isoleucine is related to the aspartate-derived pathway,^40^ which was one of the most harmaline-affected metabolic pathways (alanine aspartate and glutamate metabolism). The aspartate-derived pathway is known to be closely related to plant tolerance/detoxification mechanisms against stress.^41^

Harmaline-watered plants also experienced an accumulation of the amino acids alanine, GABA, l-aspartic acid, and proline as harmaline concentrations increased. Under abiotic stress conditions (salinity, water scarcity, nutrient deficiency/excess, or extreme temperatures), plants accumulate metabolites with an osmoprotectant function as a protective response.^42^ Among primary metabolites, amino acids are pivotal in inducing plant stress tolerance.^40^ The accumulation of alanine, glycine, proline, GABA, isoleucine, or valine has been documented to be related to osmoprotective responses to abiotic stress.^40^ Obata and Fernie^43^ showed that lysine and threonine were induced under several stress situations. In fact, Watanabe et al.^44^ demonstrated that the pool of all free amino acids significantly increases during the senescence process.

GABA’s role in plant metabolism has been investigated for being widely involved in osmoprotection, regulation of redox status, maintenance of cytosolic status, or protection against ROS like proline or glycine betaine.^45^ Significant increases in GABA concentration have already been obtained through metabolomic analysis after nerolidol treatment in *Arabidopsis* plants.^46^ In addition, during leaf senescence, GABA shunt (GABA metabolization pathway composed of three enzymes) is known to be involved in nitrogen metabolism and nitrogen fluxes that enter into the TCA cycle.^47^ Once the senescence process starts, nitrogen is converted into glutamine and asparagine, and these amino acids are transported through the phloem from senescent to young leaves to promote plant growth.^48^ Taking into account that our metabolomic analyses were done on undamaged leaves (i.e., young green leaves) after 21 days of harmaline treatment, this would be in accordance with our data, where the mobilization of molecules from senescent to young leaves in stressed plants has caused an increase in asparagine and glutamine content in harmaline-watered plants. The senescence induced by harmaline would have led to an increase in GABA content and, consequently, an increase in glutamine and asparagine concentrations due to nitrogen recycling through the GABA shunt.

On the other hand, proline is considered one of the most abundantly distributed osmoprotectants in plants, accumulated under different stress conditions in the cytosol and chloroplasts.^49^ Additionally, osmoprotectant accumulation could be related to protection from ROS, which are toxic for the cells if present at high levels.^50^ Furthermore, the primary source of proline accumulation and biosynthesis is the glutamate pathway, and our KEGG-based pathway analysis revealed that alanine, aspartate, and glutamate metabolisms were the most dysregulated pathways after harmaline treatment.

Organic acid accumulation has also been reported in our GC–MS metabolomic analysis. Specifically, citric, gluconic, and glutaric acids significantly increased their levels after harmaline treatment, whereas succinic acid was significantly lower than that in untreated plants in watering and spraying treatments. Changes in the content of different organic compounds have been related to drought tolerance because of stress conditions.^26^ Down-regulation of succinic acid could be related to the inhibition of the TCA cycle, an important pathway that provides energy for plant growth.^51^ Dysregulation of the TCA cycle, as obtained after harmaline treatment through GC–MS analysis, is known to cause plant growth reduction.^52^ In addition, Khan et al.^53^ suggested that alterations in this metabolic pathway could be related to antioxidant activities and changes in chlorophyll fluorescence, possibly causing ultrastructural alterations.

Finally, the reduction in sugar content (glucose, fructose, trehalose, and maltose, among others) after watering and spraying harmaline treatments should be highlighted. Li et al.^54^ reported a decrease in sugar content in Lanzhou lily plants after moderate and severe drought stress, suggesting that this reduction may be associated with the inhibition of photosynthesis. They also suggested that a severe deficiency of nutrients could reduce the synthesis of sugar content.^54^ Sucrose is considered a major product of photosynthesis synthesized by sucrose phosphate synthase, which means that the inhibition of photosynthesis or physical damage to the PSII could lead to reduced soluble sugar content^55^ and induce leaf senescence. Zakari et al.^32^ also suggested that a lower sugar content could be related to ROS generation and leaf-induced senescence, supporting the results obtained in this study after harmaline treatment. This is also consistent with the results obtained by Asad et al.,^56^ where decreasing concentrations of soluble sugar were related to an acceleration of leaf-induced senescence, together with an increase in ABA concentrations and ROS generation.

Our data demonstrate that harmaline is phytotoxic on adult plants of *A. thaliana* already at concentrations as low as 14 μM, inducing morphological changes and inhibiting plant growth development. Watering treatment was more effective, suggesting that it is a better way of supply instead of spraying. Harmaline-treated plants experienced leaf chlorosis, alteration on the photosynthetic machinery, and metabolomic changes, such as amino acid accumulation or reduction in sugar content. All these alterations suggest that harmaline could act by altering the plant water status and inducing early senescence, affecting the normal plant development. Harmaline might be considered as an interesting molecule for the development of botanical herbicides or to be used as a backbone for the synthesis of new herbicidal molecules with new mode of action.

## Supporting Information Available

Relative water content (RWC) information; principal component analysis (PCA) done on MS-DIAL; cross-validation and permutation test of the PLS-DA model; heat map of the significant altered metabolites after the ANOVA test on *Arabidopsis* plants; organic acid content of harmaline-watered and harmaline-sprayed plants after ANOVA analyses; data classification after testing heteroscedasticity by Levene’s test of chlorophyll *a* fluorescence parameters (PDF)
Raw metabolic data; details of metabolites identified in *A. thaliana*; details of metabolites quantified in *A. thaliana* (XLSX)
