# Supplementary material for: Application of Indole-Alkaloid Harmaline Induces Physical Damage to Photosystem II Antenna Complexes in Adult Plants of Arabidopsis thaliana (L.) Heynh
Source: J Agric Food Chem. 2023 Apr 7;71(15):6073–86. doi: 10.1021/acs.jafc.3c00531 (PMC10119982; doi:10.1021/acs.jafc.3c00531)
Supplement: Supplementary file 1 — jf3c00531_si_001.pdf [file jf3c00531_si_001.pdf]

Supporting Information

Application of indole-alkaloid harmaline induces physical damage to photosystem II' antenna complex in adult plants of Arabidopsis thaliana (L.) Heynh.

Sara Álvarez-Rodríguez[a], Carla M. Alvite[a], Manuel J. Reigosa[a], Adela M. Sánchez-Moreiras[a*], Fabrizio Araniti[b]

[a]*Universidade de Vigo, Departamento de Bioloxía Vexetal e Ciencias do Solo, Facultade de Bioloxía, Campus Lagoas-Marcosende s/n, 36310, Vigo, Spain*

[b]*Dipartimento di Scienze Agrarie e Ambientali - Produzione, Territorio, Agroenergia, Università Statale di Milano, Via Celoria nº2, 20133 Milano, Italy*

*Corresponding author: Adela M. Sánchez-Moreiras, Universidade de Vigo, Departamento de Bioloxía Vexetal e Ciencias do Solo, Facultade de Bioloxía, Campus Lagoas-Marcosende s/n, 36310, Vigo, Spain. *E-mail* address: adela@uvigo.es

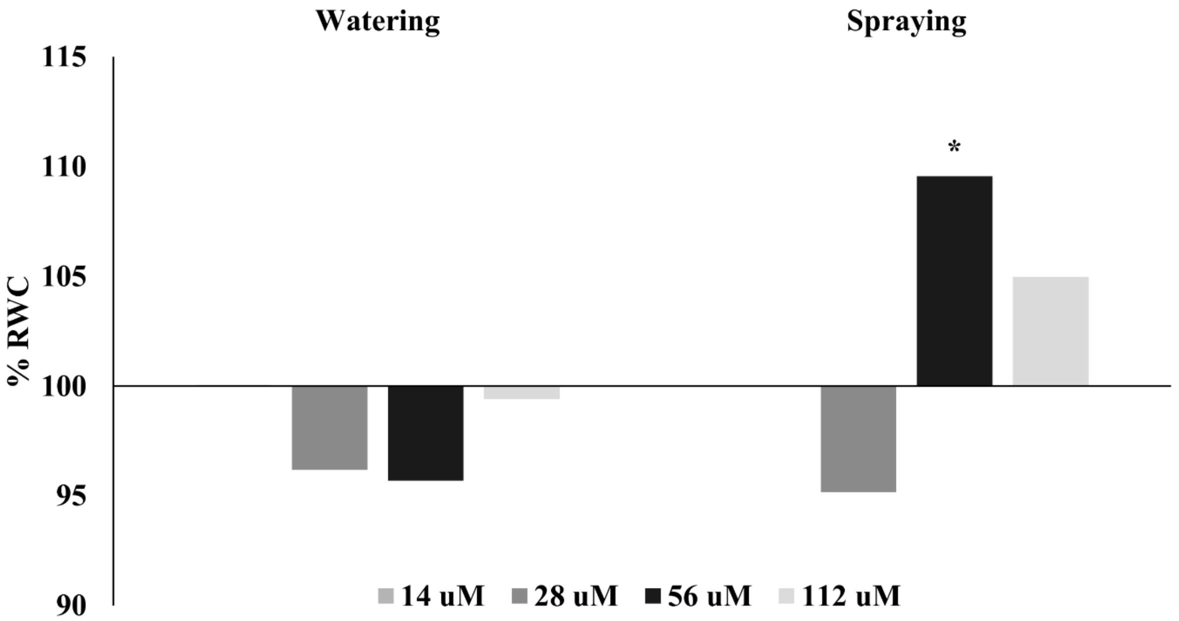

Figure S1. Relative water content (RWC) for harmaline-watered or –sprayed plants. All data are given in percentage of control. Asterisks indicate statistical differences compared to control. * $P < 0.05$, ** $P < 0.01$, *** $P < 0.001$. N=3.

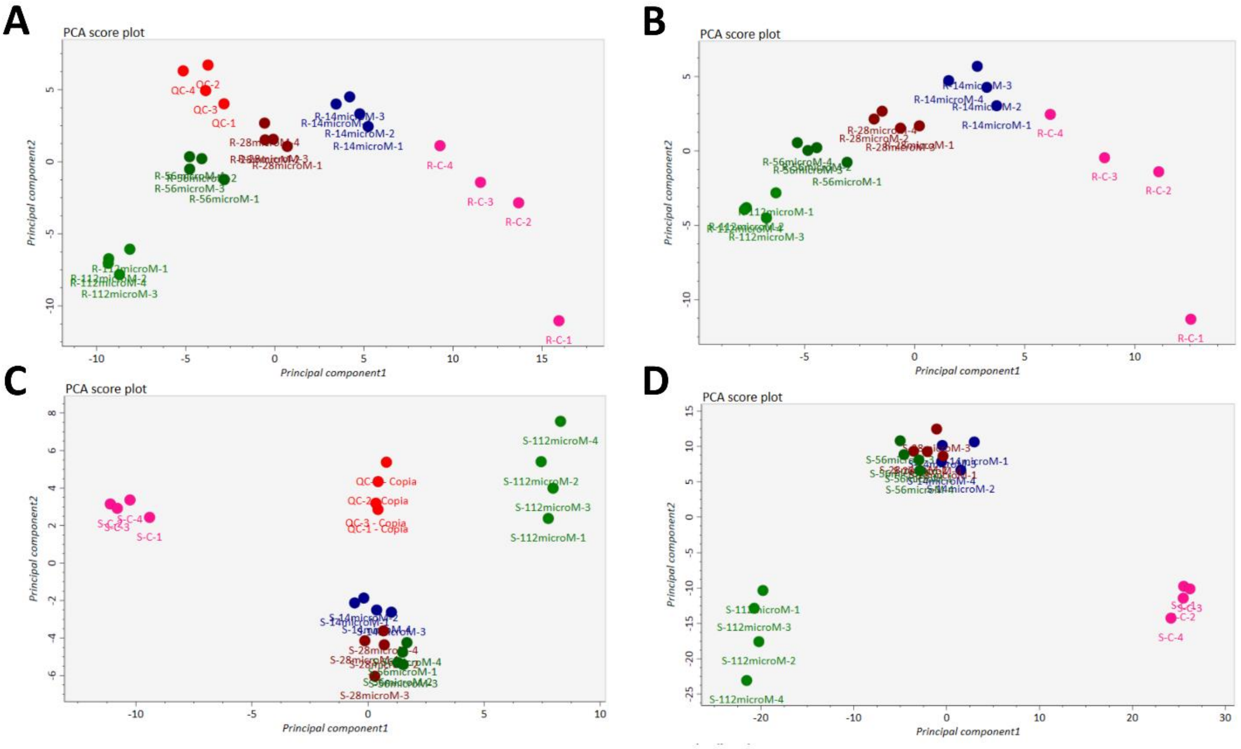

Figure S2. Discrimination through principal component analysis (PCA) done on MS-DIAL of the metabolites' patterns in *Arabidopsis thaliana* adult plants exposed for 21 d to 0, 14, 28, 56 and 112 μM of harmaline by watering and spraying. (A) and (C) PCA score plot of sample groups and qualitative control of watering and spraying, respectively; (B) and (D) PCA score plot of sample groups without qualitative control of watering and spraying, respectively . N=4.

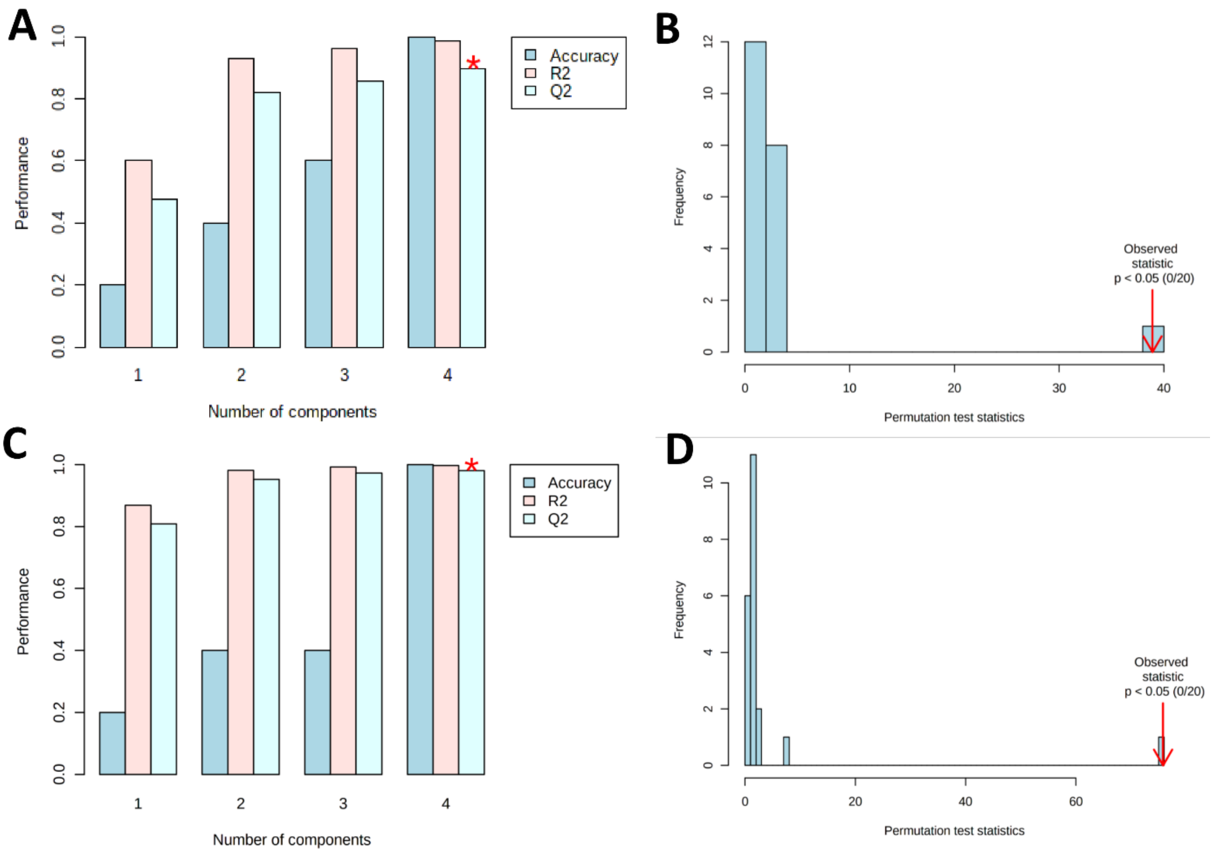

Figure S3. Cross-validation and permutation test of PLS-DA model for harmaline-watered (A and B) and harmaline-sprayed (C and D) *Arabidopsis thaliana* adult plants.

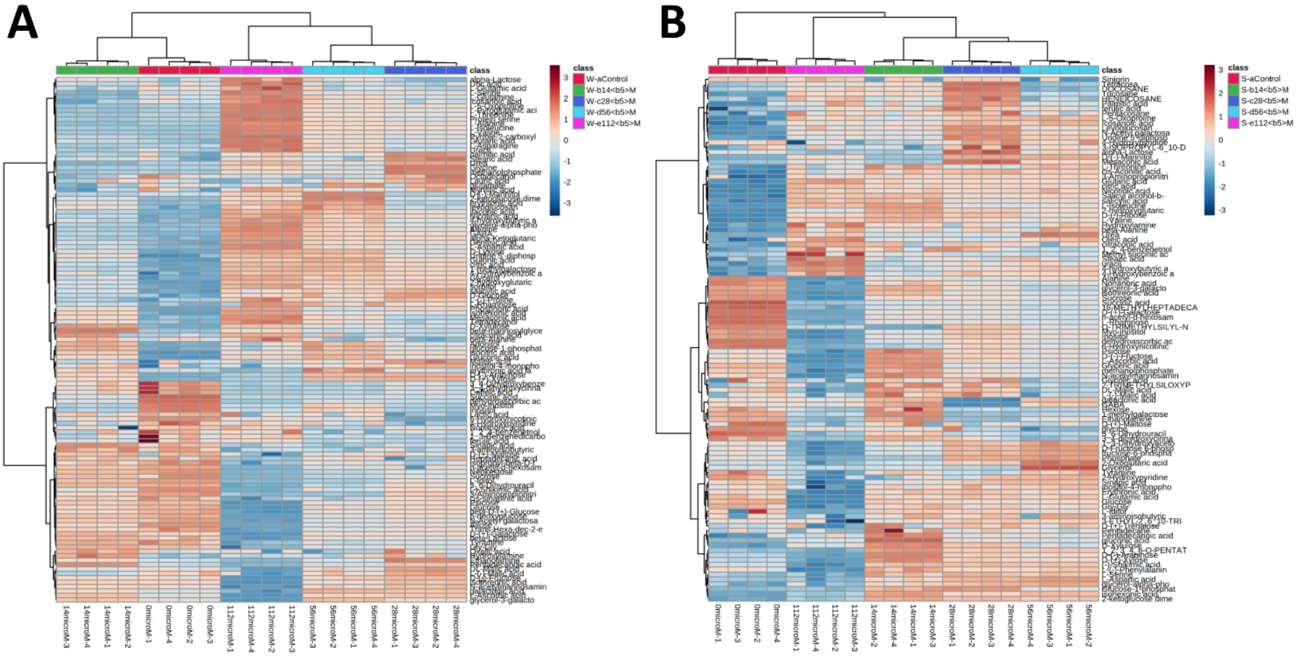

Figure S4. Overlay heat map of the 119 and 108 metabolites resulted from the ANOVA test (Tukey $P \leq 0.05$ and FDR ≤ 0.05) significantly altered in harmaline-watered (A) and harmaline-sprayed (B) *Arabidopsis* adult plants. Each square represents the effect of harmaline on the amount of every metabolite using a false-colour scale. Red and blue regions indicate an increase or decrease in metabolite content. N=4

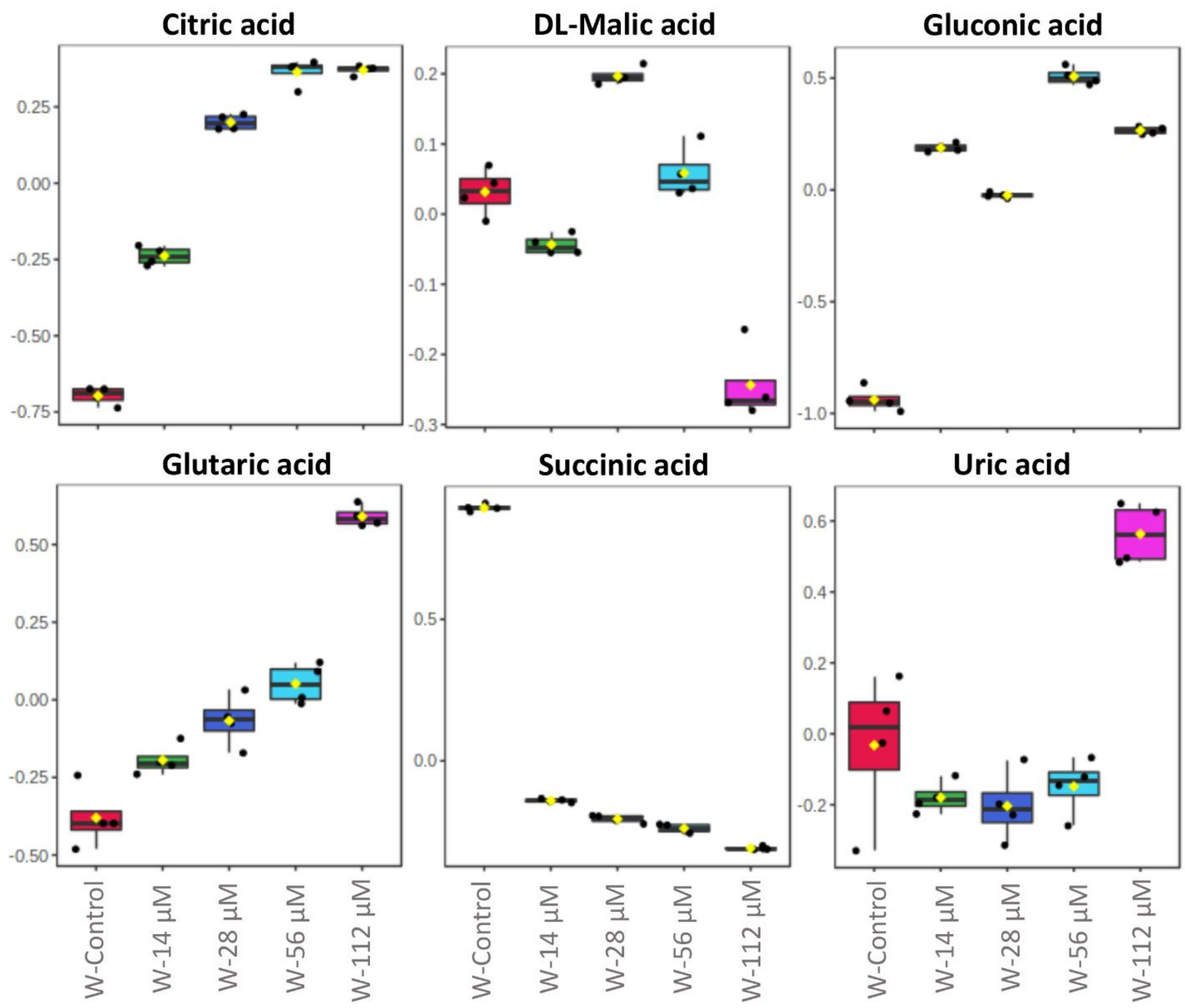

Figure S5. Effects of 0, 14, 28, 56 and 112 µM of harmaline-watered treatment on *Arabidopsis thaliana* adult plants organic acids contents. Normalised metabolomic data were analysed through ANOVA using Tukey test as pot-hoc ($p \leq 0.05$). N = 4. The full list is available on Supplementary Table S1.

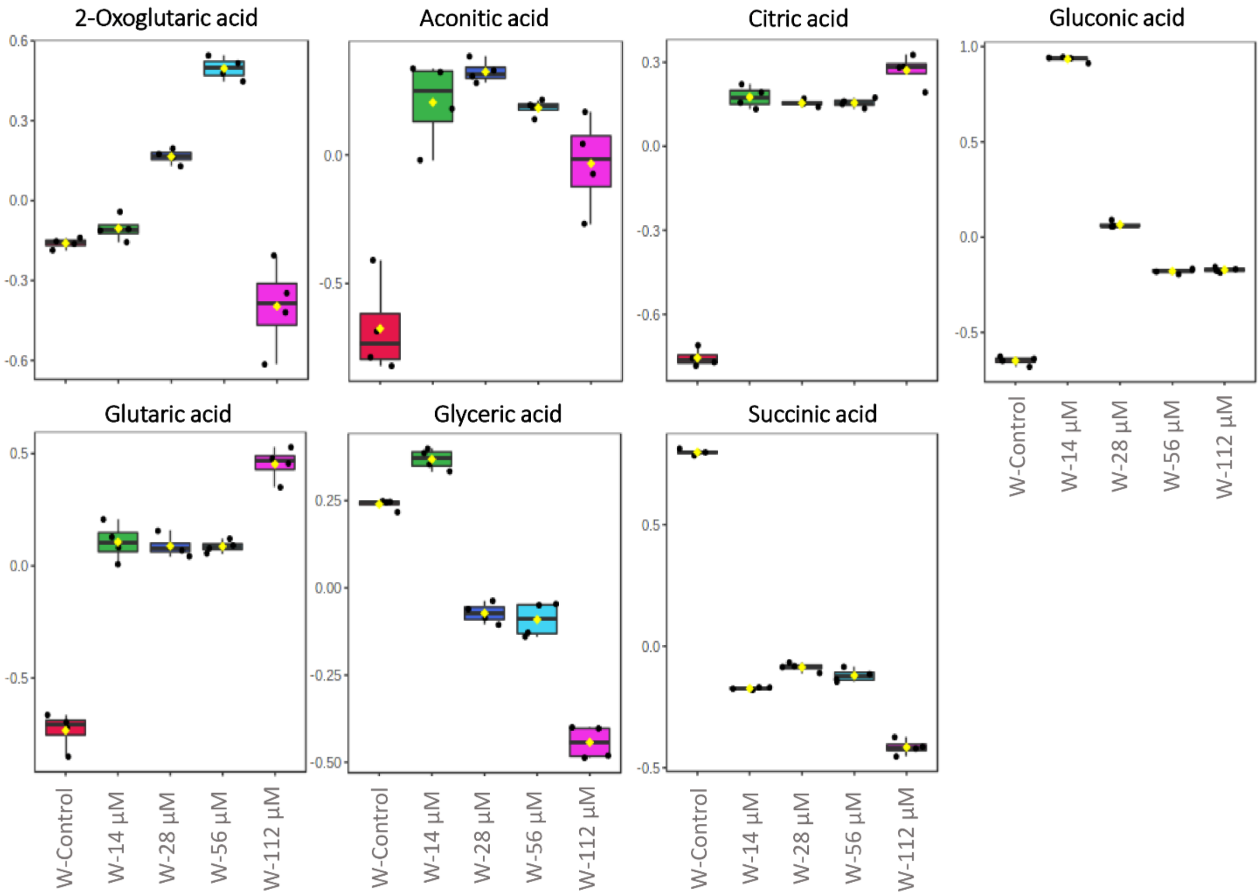

Figure S6. Effects of 0, 14, 28, 56 and 112 µM of harmaline-sprayed treatment on *Arabidopsis thaliana* adult plants organic acids contents. Normalised metabolomic data were analysed through ANOVA using Tukey test as pot-hoc ($p \leq 0.05$). N = 4. The full list is available on Supplementary Table S1.

Table S2. Data classification after testing heteroscedasticity by Levene's test of Chlorophyll *a* fluorescence parameters with SPSS Statistics 25.0. Statistically significant differences between groups were estimated by ANOVA analysis followed by the Tukey's test as post-hoc in the case of homoscedastic data and Tamhane's T2 test as post-hoc in the case of heteroscedastic data ($p \leq 0.05$).

	WATERING				SPRAYING			
Day	F_v/F_m	Φ_{II}	Φ_{NO}	Φ_{NPQ}	F_v/F_m	Φ_{II}	Φ_{NO}	Φ_{NPQ}
**0**	O	O	O	O	O	O	O	O
**2**	O	O	O	O	O	O	O	O
**4**	O	E	O	O	O	E	E	E
**7**	O	O	O	O	O	O	O	O
**9**	O	O	E	O	O	O	O	E
**11**	O	O	O	O	O	E	O	E
**14**	O	O	O	E	O	O	O	O
**16**	O	O	O	E	O	E	O	O
**18**	O	E	O	O	O	E	E	E
**21**	O	E	O	O	O	O	E	E

Homoscedastic data are reported as 'O' and heteroscedastic data are reported as 'E'.
